# Protein profiles of enzymatically isolated rumen epithelium in sheep fed a fibrous diet

**DOI:** 10.1186/s40104-019-0314-0

**Published:** 2019-01-25

**Authors:** J. J. Bond, A. J. Donaldson, J. V. F. Coumans, K. Austin, D. Ebert, D. Wheeler, V. H. Oddy

**Affiliations:** 1 NSW Department of Primary Industries, Extensive Livestock Industry Centre, University of New England, Trevenna Rd, Armidale, NSW 2351 Australia; 2 School of Rural Medicine, University of New England, Armidale, NSW 2351 Australia; 3 NSW Department of Primary Industries, Orange Agricultural Institute, Orange, NSW 2800 Australia

**Keywords:** Epithelium, Metabolism, Nutrition, Proteome, Rumen, Sheep

## Abstract

**Background:**

The rumen wall plays a major role in efficient transfer of digested nutrients in the rumen to peripheral tissues through the portal venous system. Some of these substrates are metabolised in the epithelium during this process. To identify the specific proteins involved in these processes, we used proteomic technologies. Protein extracts were prepared from ventral rumen tissue of six sheep fed a fibrous diet at 1.5× maintenance energy requirements. Using a newly developed method, we were able to enzymatically isolate the epithelial cells from underlying tissue layers, thus allowing cytosol and membrane fractions to be independently analysed using liquid chromatography tandem mass spectrometry (LC MS/MS).

**Results:**

Using our procedure we identified 570 epithelial proteins in the *Ovis aries* sequence database. Subcellular locations were largely cytosolic (*n* = 221) and extracellular (*n* = 85). However, a quarter of the proteins identified were assigned to the plasma membrane or organelle membranes, some of which transport nutrients and metabolites. Of these 91 were transmembrane proteins (TMHMM), 27 had an N-terminal signal peptide (signalP) and TMHMM motif, 13 had a glycosylphosphatidylinositol (GPI) anchor and signalP sequence, 67 had beta (β) strands or 17 β strands and a transit peptide sequence, indicating the identified proteins were integral or peripheral membrane proteins. Subunits of the 5 protein complexes involved in mitochondrial cellular energy production were well represented. Structural proteins (15%), proteins involved in the metabolism of lipids and proteins (26%) and those with steroid or cytokine action were a feature of the proteome.

**Conclusion:**

Our research has developed a procedure to isolate rumen epithelium proteins from the underlying tissue layers so that they may be profiled using proteomic technologies. The approach improves the number of proteins that can be profiled that are specific to the epithelium of the rumen wall. It provides new insights into the proteins of structural and nutritional importance in the rumen epithelium, that carry out nutrient transport and metabolism, cell growth and signalling.

**Electronic supplementary material:**

The online version of this article (10.1186/s40104-019-0314-0) contains supplementary material, which is available to authorized users.

## Background

The aim of this research was to develop a procedure to isolate rumen epithelium proteins from the underlying tissue layers so that they may be profiled using proteomic technologies. Diet induced changes in gene expression have been shown in association with genes that code proteins known to regulate the transport and metabolism of nutrients in the rumen epithelium of sheep and cattle [1, 2]. Many of these processes require multi-protein complexes and more than one or two enzymes to carry out metabolic pathways. Procedures to profile a wide range of proteins in the rumen epithelium have not been developed extensively using proteomic technologies. Hence, in our preliminary research presented here we explored our new procedures ability to improve the depth of coverage and identification of rumen epithelium proteins in relation to their function in the epithelium.

The epithelium layer is made up of four distinct cell types [3], *stratum* (*s*.) *basale*, *s. spinosum*, *s. granulosum* and *s. corneum*. These cover finger like projections called papillae. At the interface between the lamina propria (LP) and the epithelium, the *s. basale* and the *s. spinosum* are the most metabolically active. They undergo rapid cell turn over and perform tasks such as nutrient uptake, metabolic transactions and exportation into the bloodstream. Towards the surface (*s. corneum*, *s. granulosum*) of the epithelium, the cells become filled with keratins expressed in pairs (type I acidic or type II basic) that form heterodimers called intermediate filaments (IF) [4, 5]. These are crosslinked to cornified cell envelope proteins by transglutaminase enzymes [6]. The insoluble barrier formed provides protection against abrasive forces during the mixing of feed and the immune challenge [7] posed by the diverse microbial population in the rumen.

The integrity of the epithelial surface and cell-cell attachment are important in the transfer of nutrients and small molecules between the rumen lumen and basal layers of the epithelium. Proteins in desmosomes connect cell plasma membranes extracellularly whilst linking with the cells internal cytoskeleton of intermediate filaments. Electron microscopy studies reveal desmosomes are prevalent in the *s. spinosum* and *s. granulosum* [3]. Alternatively, the plasma membrane of *s. basale* cells and LP, are attached to a basement membrane by hemi desmosomes. Epithelial cells are also attached through tight junction and gap junction proteins [3].

Short chain fatty acids (SCFA) are principal nutrients in the rumen fluid [8] and their transport involves diffusion through members of the solute carrier protein family (SLC16) [9]. Glucose transport by SLC2A1 (also called SGLT-1) a Na^+^-glucose cotransporter 1 has been reported in epithelium of sheep [10]. Protein transporters for urea (SLC14A1, also called UT-B) [11] have also been identified and change with dietary nitrogen [12]. Di- and tripeptides from ingested feed or microbial protein are largely absorbed in the intestine by SLC15A1 transporter (also known as PEPT1). Although mRNA expression of *SLC15A1* has been observed in the rumen epithelium of sheep and cows [13] it has been shown to have a low flux rate and not thought to be of nutritional significance [14]. Most of these substrate transporters activity depend on concentration gradients established by membrane proteins such as the Na^+^/H^+^ antiporter (NHE) and Na^+^/K^+^-ATPase, which also aid in maintenance of pH homeostasis in the cell. In rumen epithelium, mRNA for *NHE1 NHE2, NHE3* and *NHE8*) and protein (NHE1 and NHE2) have been identified in cow [9] and mRNA and protein of Na^+^/K^+^ -ATPase detected in sheep [3, 15]. The transport activity of NHE3 has been shown in rumen tissue [16]. In addition, gene expression of transporters for glycerol (*AQP3, AQP7* and *AQP8*) [12] were identified in rumen tissue of cow, but their protein abundance was not affected by dietary treatment [17].

Nutrients are metabolised primarily in the mitochondria during the process of oxidative phosphorylation to produce cellular energy in the form of adenosine triphosphate (ATP). Mitochondria where this pathway operates are reported to be of the highest density in the *s. basale* [3]. Metabolic pathways in this cellular compartment are the site of ketone body formation such as acetoacetate and β-hydroxybutyrate. The process of ketone body formation in rumen epithelium is dependent on diet that also affects the amount of nutrients entering the bloodstream [18]. Of the three main SCFA (acetate, butyrate, and propionate) produced during microbial fermentation of plant fodder in the rumen, butyrate is a major substrate for conversion to β-hydroxybutyrate in the mitochondria and little free butyrate is recovered in venous blood from the rumen [19]. The majority of acetate is absorbed directly into the blood and some is converted to acetyl-CoA or acetoacetate in the epithelium. The propionate that is absorbed into the blood is metabolised in the liver for gluconeogenesis.

The main process of nutrient absorption is known to occur through transporters in the epithelium. Cytosol and membrane proteins play key roles in these transfers, the maintenance of intracellular homeostasis and the generation of cellular energy. Since the rumen wall is a multilayered structure (muscle, LP and epithelium), a wide dynamic range of protein abundances makes it difficult to obtain the sampling coverage required to adequately assay the complex mixture of proteins in all the tissue layers. Proteins in these specific subcellular compartments are therefore, often poorly represented when LP and epithelium is intact [20–22]. This is probably due to their relatively low abundance in relation to other subcellular locations and their hydrophobic nature. Hence we sought to improve the depth of coverage rumen epithelium proteins and relate their predicted function to their importance for animal nutrition and physiology.

## Methods

### Animals and tissue collection

Experiments were conducted in accordance with guidelines of the University of New England Animal Ethics Committee (UNE AEC approval # 14–041). Six sheep used in this study were fed a mixture of chaffed lucerne (*Medicago sativa*) and oaten hay (*Avena sativa)* 50:50 *w*/*w* (Manuka feeds Quirindi, Australia) for a 4-week period prior to slaughter and tissue collection. The feed composition is described in detail in [23] and was composed of 90% dry matter (DM), 16.3% crude protein (CP), 50.6% neutral detergent fibre (NDF) and 9.9 megajoules of metabolisable energy per kilogram of dry matter (MJ ME /kg DM). Each ewe was dry, non-pregnant, non-lactating, 3 years old and fed in individual pens at 1.5× maintenance requirements of ME for their live weight calculated according to Australian feeding standard guidelines [24]. Feed was provided twice daily, with half the daily ration offered at 08:00 h and the other half at 16:00 h. Drinking water was available at all times. At the end of the experiment sheep were humanely slaughtered by a qualified operator. Samples of whole depth rumen wall from the ventral sac were removed and rinsed with phosphate buffered saline (PBS) to remove plant matter. The tissue was snap frozen in liquid nitrogen approximately 10 min after stunning and slaughter and stored at − 80 °C till further preparation of the epithelium for protein separation.

### Preparation of isolated epithelium

Each frozen tissue piece from 6 sheep was cut with a scalpel into approximately 2 mm wide strips. The muscle layers were mechanically sheared from the epithelium and LP using forceps under a dissecting microscope (M3Z, Wild Heerburg, Switzerland) and discarded. Remaining tissue strips were washed in cell wash solution (MemPer™ Plus membrane kit, Thermo scientific, IL, USA) and placed in a solution of 1.2 U/mL Dispase II in phosphate buffered saline (PBS) for 3 h, 8 h and overnight at 4 °C. Following this, sheets of epithelium were separated from the LP under the dissecting microscope and samples of epithelium frozen at − 20 °C. The overnight treatment was required for easy separation of tissue by microdissection, thus all subsequent enzymatic digestions were conducted overnight (~ 18 h) at 4 °C.

### Histology of rumen wall tissue

Whole depth rumen wall tissue and enzymatically separated epithelium and LP were embedded in Tissue-Tek® O.C.T compound (Sakura, CA, USA), snap frozen in liquid nitrogen and cryostat (cryotome SME, Life sciences, Cheshire, UK) sectioned (10 μm). Sections were stained in Wiegert’s iron haematoxylin solution (Sigma, MO, USA) for 6–8 min, and then rinsed in tap water to clear unbound stain. Sections were then immersed in Gomori trichrome stain (Sigma, MO, USA) for 10 min, and the colour differentiated with 0.2% acetic acid. Sections were then dehydrated in ascending ethanol 95–100% for 2 × 5 min each change of solution. The sections were then cleared with xylene substitute (Sigma, MO, USA) for 3 × 5 min. A coverslip was mounted with DPX solution (Sigma, MO, USA), and air dried overnight.

### Homogenisation and extraction of epithelial cytosol and membrane proteins

A wet weight approximately 0.5–1 g of individual samples of epithelium was placed in a 2-mL stainless steel tube with 1 mm stainless steel beads (0.2 g, Daintree scientific, St Helens, Tas, Australia). One mL of cell permeabilisation buffer (memPer™ Plus membrane kit, Thermo scientific, IL, USA) and 20 μL of protease inhibitor (Sigma, MO, USA) were added and tube incubated for 20 min at 4 °C. Tissue was homogenized by bead beating for 8 × 30 s and put on ice for 30 s between each shake at 4,800 oscillations per min (Biospec products, OK, USA). To remove cell debris, the homogenate was centrifuged at 900 *× g* for 5 min (Mini spin, Eppendorf, Hamburg, Germany). The supernatant was then placed in a clean tube and centrifuged at 16,000 *× g* for 15 min at 4 °C (Universal 32R, Hettich Tuttlingen, Germany). The supernatant containing the cytosolic proteins was carefully removed and dialysed in a mini dialysis device with a 3.5-dalton (Da) molecular weight cut off (MWCO) membrane (Spectra/Por® 3, Spectrum laboratories, CA, USA). The supernatant was equilibrated to 0.1 mol/L tris (hydroxymethyl) aminomethane (Tris) pH 8.5 with 3 changes of buffer over 18 h.

The remaining pellet was suspended in 1 mL of membrane solubilisation buffer (memPer ™ Plus membrane kit, Thermo scientific, IL, USA) with sonication for 5 s at 250 watts (Labsonic probe 1510, Braun, Germany). The suspension was incubated for 30 min at 4 °C with constant mixing then centrifuged at 16,000 *× g* for 15 min at 4 °C. The supernatant containing solubilized membrane and membrane associated proteins was dialysed in a mini dialysis device with 3.5 Da MWCO dialysis membrane in 3 changes of 5% sodium dodecyl sulfate (SDS) in 0.1 mol/L Tris pH 8.5, over 18 h. Protein concentration of cytosol proteins was quantified by a Pierce™ BCA protein assay (Thermo scientific, IL, USA) and membrane proteins using the 2D Quant Kit (GE Healthcare, NJ, USA).

### Membrane protein separation

Membrane protein was solubilised in 1× NuPAGE® LDS sample buffer (0.025 mol/L Tris pH 8.5, 0.5% LDS, 2.5% glycerol, 0.05 mol/L dithiothreitol (DTT), 0.05 mol/L SERVA Blue G250, 0.04 mmol/L phenol red). Fifty μg of protein from each sheep was loaded in duplicate lanes on Bolt 4–12% Bis-Tris gels. Proteins were separated in a NuPAGE® MOPS running buffer system (Invitrogen, CA, USA) at a constant 200 V using a mini gel tank (Life technologies, CA, USA). Gels were stained with 0.1% coomassie brilliant blue (CBB R250) in 50% methanol (Chem–supply, SA, Australia) and 10% glacial acetic acid (Chem–supply, SA, Australia), then destained in 40% methanol and 10% glacial acetic acid. Gel images were obtained using a gel doc system (BioRad, CA, USA). Each gel lane was then cut into 9 pieces prior to protein digestion with trypsin and elution from gel piece.

### Sample preparation LC-MS/MS analysis

Proteins of the cytosolic fraction (15 μg) were prepared for LC-MS/MS as previously described [25]. Briefly, proteins were reduced using tris (2-carboxyethyl) phosphine (0.0025 mol/L, 60 °C, 60 min), alkylated with iodoacetamide (0.002 mol/L, 20 °C, 10 min) and digested with trypsin (0.5 μg/50 μg protein) (Sigma NSW, Australia) for 16 h at 37 °C. Following trypsin digestion, peptides were purified using Pierce® C18 spin columns (Pierce Biotechnology, IL, USA) following the manufacturer’s instructions. Membrane proteins from preparative gels were destained and digested as previously described [26].

### Mass spectrometry

Peptides were separated using a Prominence nanoflow-LC (Shimadzu, Kyoto, Japan). Samples (2–7 μL) were concentrated and desalted on a ChromXP C18 pre-column (350 μm × 0.5 mm, Eksigent) with water: acetonitrile (H_2_O:CH_3_CN, 98:2, 0.1% formic acid). After a 5-min wash the pre-column was automatically switched into line with an analytical column (75 μm × ~ 12 cm) containing C18 reverse phase packing material (Magic, 5 μm, 200 Å). Peptides were eluted using a linear gradient of H_2_O:CH_3_CN (95:5, 0.1% formic acid) to H_2_O:CH_3_CN (60:40, 0.1% formic acid) at ~ 200 nL/min over 35 min for membrane proteins previously separated by SDS-PAGE and 60 min for proteins found in the cytosolic fraction. The analytical C18 column connected via a fused silica capillary (10 cm, 25 μm) to a PicoTip Emitter (New Objective, MA, USA) where high voltage (2,300 V) was applied and the column tip positioned ~ 1 cm from the Z-spray inlet of a TripleTOF 5600 MS aaasaaa(ABSciex, MA, USA). Positive ions were generated by electrospray and the TripleTof operated in information dependent acquisition mode (IDA). A time of flight (Tof) MS survey scan was acquired (350–1,500 mass (m)/charge (z), 0.25 s) and the multiply charged ions (2+ to 5+) in the 400–1,250 m/z range were sequentially selected by quad one (Q1) for MS/MS analysis. Nitrogen was used as collision gas and an optimum collision energy chosen (based on charge state and mass). Tandem mass spectra were accumulated for up to 0.1 s (100–2,000 m/z).

### Database searching

The calibrated experimental peptide masses or MS/MS average mass data files were imported into MASCOT [27] for searches against the Uniprot mammalia database (234,491 sequences and 90,265,790 residues Uniprot 20.06.2016). The matches to protein sequence are based on m/z ratios. For positive identification of proteins search mass tolerance parameters were set at two missed cleavages per peptide, modification of the cysteine by carbamidomethylation or methionine by oxidation and a protein mass tolerance of 1.5 Da and a peptide mass tolerance of 0.5 Da, with 2+ and 3+ peptide charge states. Search match stringencies to obtain a correct identification included a total protein (*P <* 0.05) and individual peptide score (*P <* 0.05) for at least two peptides, which were found in duplicate samples and protein extracts from each of the 6 sheep analysed. Alternatively MS/MS mass data files (*.wiff) were imported into PEAKS studio software [28] for searches against the ovine Ensembl database (Oar_3.1pep.fasta Ensembl 21.05.2017). For MS/MS identifications search tolerances used were two missed cleavages per peptide, modification of the cysteine by carbamidomethylation or methionine by oxidation and a protein mass tolerance of 20 ppm and a peptide mass tolerance of 0.1 Da. Search match stringencies included a de novo average local confidence (ALC) score ≥ 50%, false discovery rate (FDR peptide-spectrum matches) at 2.0%, protein score − 10log*P* ≥ 20, at least two unique peptides matches per protein, with a sequence coverage > 3%, in the 6 biological replicates.

### Bioinformatics

Additional annotation information was provided by cross-referencing Ensembl ovine database identifications to the Uniprot mammalia database. Subcellular location and functional assignment of ovine proteins was based on gene ontology description, as well as domain information from the Uniprot annotations. In addition, these were cross checked with subcellular location based on sequence specific information from the Ensembl ovine database using the top score generated using WoLF PSort website [29].

Membrane protein prediction was based on TMHMM, β strand, GPI anchor, N-terminal signal peptide cleavage sites (SigP) and transit peptide domains. Proteins with one or more transmembrane domains were determined with THMHH server v 2.0 [30] and SignalP using 4.1 sever [31]. Those with β strand associated with β barrel proteins were determined from information in Uniprot mammalia annotations. Glycosylphosphatidylinositol (GPI) anchor domains were predicted using big-PI predictor [32].

## Results

### Sample preparation development

As tissue with the epithelium and LP combined required considerable mechanical force to homogenise; grinding with a mortar and pestle in liquid nitrogen was required. In these initial extracts, the total concentration of the protein was relatively low and the proteins identified were mainly albumin and other high abundance blood related proteins.

**Fig. 1:**
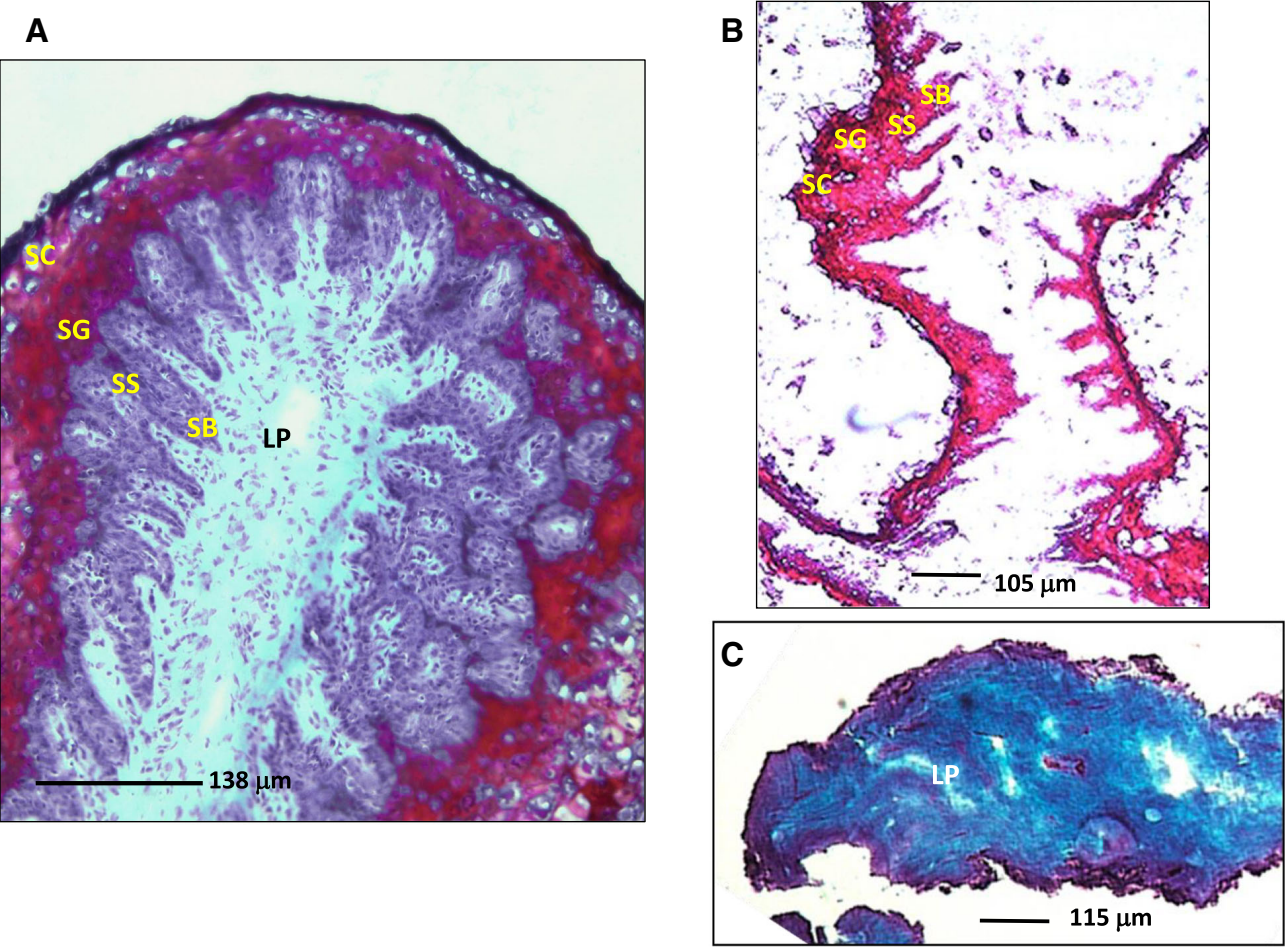
Panel **a**. A representative tissue section of the tip of a rumen papillae magnified with a 10× objective using a light microscope and stained with trichrome. The lamina propria (LP) is stained aquablue, *s. basale* (SB) and *s. spinosum* (SS) is stained purple and outer *s. granulosum* (SG) and *s. corneum* (SC) stained maroon. Panel **b**. Epithelium tissue isolated enzymatically stained a purple/maroon colour and layers labelled as above. Panel **c**. Isolated lamina propria tissue stained an aquablue – light blue colour after being separated from the epithelium. Scale in μm is drawn on each plate

Using enzymatic digestion to separate the layers, isolated epithelium and coverage of epithelial specific and membrane proteins was enriched. Upon dissection the epithelium was removed as entire sheets or pieces from the underlying LP and required further bead beating to homogenise the intact cells. An example of the epithelium removed from one isolated papillae is shown by histological section and trichrome staining in Fig.1. As shown only a few epithelium cells remained attached to the LP indicating the separation of the layers occurred in the basement membrane. The specificity of the enzyme [33, 34] allowed us to isolate the epithelium layer from the LP without undue force to reduce tearing. Technical variation between samples due to the dissection procedure was accounted for by biological and technical replication described in the methods.

The procedure required a step to remove substances in the ‘memPER’ extraction procedure that interfered with accurate quantification of the total protein concentration. Several precipitation procedures were attempted. However, dialysis proved the most effective and convenient approach. This allowed reasonable resolution and separation of membrane proteins by gel electrophoresis, without introducing quenching of the spectral intensity during analysis by MS/MS.

Following fractionation of isolated epithelium using the ‘memPER kit’ procedure we were able to obtain a cytosol and a membrane fraction. From gene expression studies [1] on similar tissue the majority of the proteins predicted to be in the epithelium cluster had a theoretical weight between 200 and 10 kDa. To obtain coverage of membrane proteins in this size range, 4–12% gradient Bis-Tris gels were used. This provided the best resolution of proteins in the epithelium membrane fraction compared to other gel gradients and buffer systems trialled. In total, we identified 570 proteins using this sample preparation procedure.

### Subcellular location of epithelium proteins

**Fig. 2:**
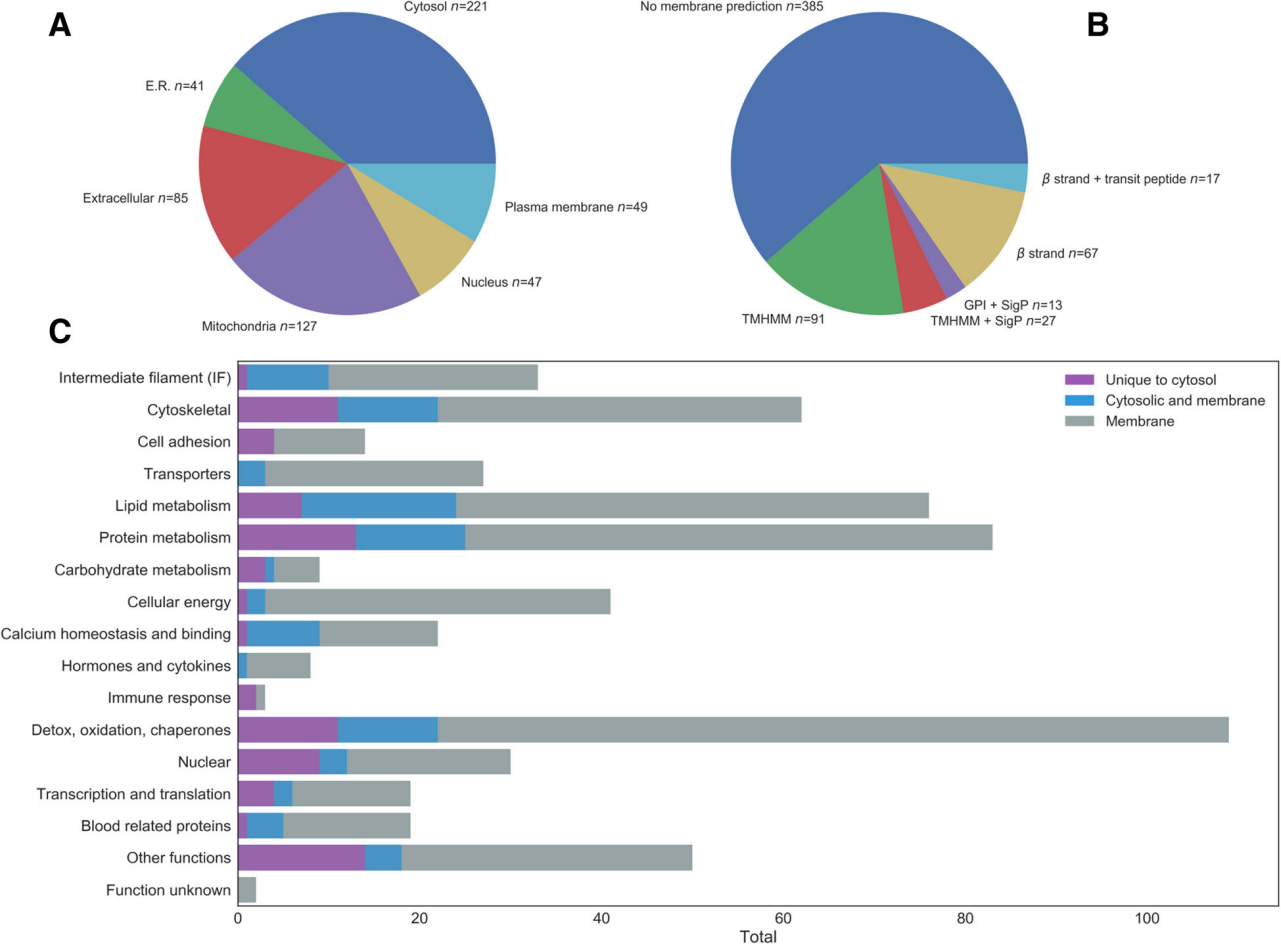
The following graphs represent the proteins identified using the Ensembl *Ovine aries* database from rumen epithelial tissue of 6 sheep. Panel **a**. A pie diagram of the distribution of subcellular compartment assigned to proteins identified. Panel **b**. A pie chart to show the proportions of predicted membrane proteins. Panel **c**. The biological function of proteins identified in the rumen epithelial cytosol and membrane fraction

The predicted subcellular location of epithelium proteins in the combined cytosol and membrane fraction is shown in Fig. 2a. More than half of the proteins identified had a subcellular location in the cytosol (*n* = 221) or extracellular (*n* = 85) compartment. Around a quarter were predicted as plasma membrane (*n* = 49) or associated with membranes of organelles such as mitochondria (*n* = 127) endoplasmic reticulum (ER; *n* = 41) or nucleus (*n* = 47).

Analysis showed that 215 were predicted to be membrane proteins (Fig. 2b). These include 91 proteins with a transmembrane domain (TMHMM), 67 with a β strand associated with β barrel proteins and 13 with a GPI anchor and N-terminal signalP. In addition, 27 had a TMD and SignalP associated with the secretion of proteins in association with a cell membrane. β strand and a transit peptide sequence related to location in the mitochondria were found in 17 proteins.

### Gene Ontology (GO) terms for biological functions of epithelial proteins

The predominant functions of the proteins found in the cytosol fraction (Fig. 2c) were intermediate filaments (IF) and other cytoskeletal proteins (15%). Seven percent were involved in metabolite transport and cell attachment, 12% lipid metabolism or 13% protein metabolism whereas only 1% was involved in carbohydrate metabolism. Many proteins involved in producing cellular energy were identified (7%). Other groups of proteins found were those that play a role in the regulation of calcium (Ca^2+^) homeostasis or binding (4%), detoxification, oxidation and chaperones (18%). Other categories of proteins included those involved in processes in the nucleus, transcription or translation (8%), proteins found in blood (3%) or with uncharacterised function (10%).

### Intermediate filaments and associated proteins

Throughout the vertebrate body, epithelium may be classified according to its morphology and spatial expression pattern of keratins. The location of keratin expression changes from the basal layers to the corneum during differentiation and has been modelled in stratified epithelium, skin. We found evidence that the rumen is a stratified epithelium with keratin expression found in skin. Pairs such as KRT5 and KRT14 usually found in the *s. basale* at the start of differentiation were identified. Keratins associated with basal or suprabasal layers including 17 type II and 12 type I soft epithelial keratins were identified (Table 1). Keratins associated with the late cornified envelope (LCE) on the surface or in the *s. granulosum* and *s. corneum* included 4 type II and 7 type I hard epithelial keratins usually found in hair, hoof or horn. Four keratin associated proteins (KAP) and 1 small proline-rich protein were also identified. Transglutaminase 3 (TGM3) was the main enzyme identified that acts to cross-link the IF in the surface layers. Of those known to regulate IF signalling and remodelling, we found 14–3-3 proteins. Other cytoskeletal proteins identified may be found in Additional file 1.

**Table 1:** Type I and II keratins, intermediate filaments and KAPs identified in the rumen epithelium

Uniprot No.	Ensemble No.	Protein name	Gene name	Av. M*r,* kDa	−10log*P*	Pep.	Cov., %
Soft epithelial keratins							
W5Q611	ENSOARP00000018151	Keratin 1	*KRT1*	75.8	133.62	12	12
W5Q687	ENSOARP00000018227	Keratin 5	*KRT5*	62.7	281.92	49	66
W5Q6B8	ENSOARP00000018258	Keratin 6B	*KRT6B*	57.3	299.79	63	84
W5Q6E8	ENSOARP00000018288	Keratin 75	*KRT75*	54.2	187.5	27	24
W5Q5Z3	ENSOARP00000018133	Keratin 77	*KRT77*	60.8	109.78	14	8
W5Q5Q3	ENSOARP00000018043	Keratin 78	*KRT78*	57.5	114.75	12	8
W5Q5S8	ENSOARP00000018068	Keratin 79	*KRT79*	56.5	174.18	18	15
W5Q6V9	ENSOARP00000018450	Keratin 80	*KRT80*	48.1	65.27	9	3
W5Q160	ENSOARP00000016446	Keratin 10	*KRT10*	57.3	182.7	17	18
W5Q665	ENSOARP00000018205	Keratin 13	*KRT13*	49.7	215.59	42	26
W5Q6L8	ENSOARP00000018359	Keratin 14	*KRT14*	48.6	266.74	66	43
W5Q6A4	ENSOARP00000018244	Keratin 15	*KRT15*	49.6	207.33	45	26
W5Q702	ENSOARP00000018494	Keratin 16	*KRT16*	41.1	163.74	12	11
W5Q744	ENSOARP00000018536	Keratin 17	*KRT17*	50.4	299.81	68	64
W5Q5M3	ENSOARP00000016006	Keratin 24	*KRT24*	48.5	135.28	12	9
Hard epithelial keratins							
W5Q6M7	ENSOARP00000018368	Keratin 83	*KRT83*	50.3	135.98	20	8
W5Q0H4	ENSOARP00000016210	Keratin 25	*KRT25*	49.4	155.37	8	10
W5Q0U7	ENSOARP00000016333	Keratin 27	*KRT27*	50.1	86.13	10	6
W5Q0V6	ENSOARP00000016342	Keratin 28	*KRT28*	50.8	80.98	12	6
W5Q5A7	ENSOARP00000017897	Keratin 32	*KRT32*	45.1	111.31	14	9
W5Q5N5	ENSOARP00000018025	Keratin Ha5	*KRT35*	50.5	143.53	30	14
W5Q5X4	ENSOARP00000018114	Keratin 36	*KRT36*	51.4	256.92	66	46
Keratin associated and small proline rich proteins							
J9SVG9	ENSOARP00000022818	Keratin associated protein 6-L	*KAP6-L*	7.3	90.99	41	2
F5AY94	ENSOARP00000008484	Keratin associated protein 11–1	*KRTAP11–1*	16.9	83.69	34	4
W5NRL7	ENSOARP00000000807	Keratin associated protein 15–1	*KRTAP15–1*	14.7	75.17	19	2
W5NPI9	ENSOARP00000000079	Keratin associated protein	*KAP*	14.2	94.09	31	3
W5NRP7	ENSOARP00000000837	Keratin associated proteins.	*KRTAP, PMG*	17.5	88.98	18	3
W5QJB0	ENSOARP00000022817	Small proline-rich protein	*SPRR*	7.0	82.07	35	3
W5P9A6	ENSOARP00000007014	Small proline-rich protein	*SPRR*	7.8	82.07	31	3
W5QJB7	ENSOARP00000022821	Small proline-rich protein	*SPRR*	7.9	82.07	31	3

Uniprot mammalia and Ensembl ovine accession number, protein name, gene nomenclature, average molecular weight (kDa), PEAKS software probability score (− 10log*P*), number of peptides matched (Pep.) and sequence coverage (Cov. %) are tabulated

### Transporter proteins

A summary of proteins and metabolite transporters found by mRNA or using the procedure described here in the same tissue are listed in Table 2 and Additional file 1. Those transporters requiring ATP for active transport were all related to functions on the cell surface such as Na^+^/ K^+^ transporting ATPase subunits (ATP1A1, ATP1B1) and one ATP-binding cassette transporter (ABCC3). Carbonic anhydrase (CA1) and a Cl^−^ /HCO_3_^−^ exchanger (SLC26A3) which both plays roles to maintain intracellular pH and the diffusion gradient across the plasma membrane for SCFA uptake were detected. In addition, selenium-binding protein 1 was identified (SELENBP1). Other members of the SLC family of antiport, uniport and exchange transporters found were related to transport of amino acids (SLC25A11, SLC25A13) and ion homeostasis (SLC25A3, ATP2A2) within the cells mitochondria and ER.

**Table 2:** A summary of transporters identified by mRNA in whole rumen epithelium [17] or protein (bold) identified in enzymatically isolated epithelium from the same tissue by LC MS/MS [16]

Uniprot No.	Ensembl No.	Gene name	Synonym	Subcellular location	Identification type
Nutrient absorption and transport					
W5QET6	ENSOARG00000019772	*SLC16A1*	MCT1	Plasma membrane	RNA
P53791	ENSOARG00000011955	*SLC5A1*	Na^+^glucose cotransporter 1	Plasma membrane	RNA
Inorganic ion transport and exchange					
W5Q2U6	ENSOARG00000015862 ENSOARP00000017034	*SLC9A2* *SLC9A3*	NHE, Na^+^/H^+^ exchanger	Plasma membrane	RNA
**W5PAD4**	**ENSOARP00000007393**	*SLC26A3*	Cl^−^/bicarbonate exchanger	Plasma membrane	Protein
**W5PZ07**	**ENSOARP00000015693**	*CLCA2*	Cl^−^ channel accessory 2	Plasma membrane	Protein
**P04074** **P05028**	**ENSOARP00000021706** **ENSOARP00000010054**	*AT1A1* *AT1B1*	Na^+^/K^+^ ATPase transporting subunits	Plasma membrane	Protein
**W5QIK8**	**ENSOARP00000022560**	*SELENBP1*	Selenium binding protein 1	Plasma membrane	Protein
Metabolite transport across organelle membrane					
**W5P061**	**ENSOARP00000003809**	*ABCC3*	ATP binding cassette subfamily C	Plasma membrane	Protein
**B2MVX2**	**ENSOARP00000006141**	*SLC25A11*	Oxoglutarate carrier	Mitochondrial	Protein
**W5P1S3**	**ENSOARP00000004372**	*SLC25A13*	Aspartate/glutamate carrier	Mitochondrial	Protein
**W5PTW2**	**ENSOARG00000012970**	*SLC25A3*	Phosphate carrier		Protein
**W5PCT5**	**ENSOARP00000008247**	*SLC25A6*	Adenine nucleotide translocator	Mitochondrial	Protein
**W5PBQ7**	**ENSOARP00000007868**	*SLC25A50*	Mitochondrial carrier	Mitochondrial	Protein
W5PPK8	ENSOARG00000011554	*SLC30A1*	Calcium transport	Membrane	RNA
**W5Q3Y8**	**ENSOARP00000017427**	*ATP2A2*	Calcium-transporting atpase	Endoplasmic reticulum	Protein
W5NZ03	ENSOARG00000003192	*SLC33A1*	Acetyl-coa transmembrane transporter	Membrane	RNA
W5Q8V9 W5PQ03	ENSOARG00000017849 ENSOARG00000011685	*SLC35A3* *SLC35F5*	Pyrimidine nucleotide-sugar transporter	Golgi	RNA
W5P739	ENSOARG00000005820	*SLC35B1*	UDP-galactose transporter	Endoplasmic reticulum	RNA
W5PTA4	ENSOARG00000012780	*SLC37A3*	Sugar/phosphate exchange	Endoplasmic reticulum	RNA
**W5PW62** **W5PG36** **W5NZM0**	**ENSOARP00000014695** **ENSOARP00000009401** **ENSOARP00000003618**	*VDAC1* *VDAC2* *VDAC3*	Voltage dependent anion channel	Mitochondrial	Protein

Accession number for Uniprot and Ensembl *Ovine aries* database, gene name, synonym, subcellular location, and identification status are found in the Table below. Details of the protein identifications are found in Additional file 1

### Proteins associated with cell-cell attachment

Those proteins involved in cell-cell attachment (Additional file 1) to the plasma cell membrane via desmosomes included desmocollin (DSC2 and DSC3), desmoglien (DSG3), desmoplakin (DSP) and junction plakoglobin (JUP). Proteins found in the basement membrane and hemi desmosomes were fibronectin (FN1), lamin (LMNA) and 7 integrins (ITGA2, ITGA3, ITGA6, ITGAV, ITGB1, ITGB4, and ITGB5) and one proteoglycan (heparan sulfate proteoglycan; HSPG2). Others that play a role in cell adherence via actin filaments include catenin (CTNNA1and CTNNB1) and cadherin (CDH1 and CDH13).

### Proteins that metabolise lipids or proteins

The transport of long chain FA into the mitochondria for β-oxidation is accomplished by the carnitine palmitoyltransferase system (CPT1, SLC25A20 and CPT2). We identified carnitine O-palmitoyltransferase 2 (CPT2) of this shuttle (Additional file 1). Other proteins associated with lipid homeostasis or cholesterol metabolism and transport identified included liver carboxylesterase 1.

Proteins that play a pivotal role in the synthesis and degradation of SCFA to ketone bodies from acetyl CoA were detected. These include short-chain specific acyl-CoA dehydrogenase (ACADS), hydroxymethylglutaryl-CoA synthase (HMGCS2), 3-ketoacyl-CoA thiolase (ACAA2) and D-β-hydroxybutyrate dehydrogenase (BDH1).

Phospholipids are a major component of cell membranes, a substrate for arachidonic acid metabolism and the synthesis of prostaglandin and leukotrienes. The identified proteins involved in arachidonic acid metabolism and prostaglandin metabolism include arachidonate lipoxygenase (ALOX15B), leukotriene A4 hydrolase (LTA4H) and prostaglandin reductase (PTGR1).

We also identified a less well-known protein adipocyte plasma membrane-associated protein (APMAP) usually associated with adipocyte metabolism. The protein has been shown to exhibit strong aryl esterase activity, which is associated with high- density lipoproteins (HDL) and fatty acid transport in the blood and may protect low- density lipids (LDL) from oxidation. Two fatty acid binding proteins were also found (FABP4 and FABP5).

We identified several proteolytic enzymes some of which were part of larger proteolytic apparatus in the cell that can hydrolyse proteins and peptides into free amino acids (Additional file 1). These included 11 subunits of the 20S core proteasome complex (proteasome subunit alpha or beta type), mitochondrial aspartate aminotransferase (GOT2) and glutamate dehydrogenase, lysozyme C, cathepsin C and two subunits of amine oxidase (flavin containing subunit A and B).

### Proteins of the electron transport chain apparatus

Enzymes identified from the citric acid cycle are shown in Additional file 1. The reduced nicotinamide adenosine dinucleotide (NADH) generated in the citric acid cycle can feed the electron transport chain apparatus during oxidative phosphorylation to provide cellular energy, in the form of ATP. The apparatus is composed of 5 multiprotein complexes of the inner membrane of the mitochondria of which we identified 40 proteins in rumen epithelium (Table 3).

**Table 3:** A summary of the 5 multiprotein complexes that function in oxidative phosphorylation identified in rumen epithelium

Uniprot No.	Ensembl No.	Gene name	Protein name	-10log*P*	Pep.	Cov., %
Complex I- NADH dehydrogenase (EC 1.6.5.3)						
W5QB34	ENSOARP00000019929	*NDUFS1*	NADH-ubiquinone oxidoreductase 75 kDa subunit	200.39	21	13
W5PJ73	ENSOARP00000010493	*NDUFS2*	NADH dehydrogenase [ubiquinone] iron-sulfur protein 2	30.01	6	2
W5 PB27	ENSOARP00000007638	*NDUFS3*	NADH dehydrogenase [ubiquinone] iron-sulfur protein 3	140.41	32	9
W5PUX0	ENSOARP00000014252	*NDUFV1*	NADH dehydrogenase [ubiquinone] flavoprotein 1	91.04	14	8
W5NRY1	ENSOARP00000000921	*NDUFV2*	NADH-ubiquinone oxidoreductase core subunit V2	113.11	27	6
W5QAH8	ENSOARP00000019723	*NDUFA2*	NADH dehydrogenase [ubiquinone] 1 alpha subunit 2	85.83	31	3
W5PYA5	ENSOARP00000015439	*NDUFA8*	NADH dehydrogenase [ubiquinone] 1 alpha subunit 8	124.37	30	5
W5QBF5	ENSOARP00000020052	*NDUFA10*	NADH dehydrogenase [ubiquinone] 1 alpha subunit 10	45.28	9	2
W5QHN8	ENSOARP00000022238	*NDUFB5*	NADH dehydrogenase [ubiquinone] 1 beta subunit 5	86.49	14	2
W5PZE3	ENSOARP00000015829	*NDUFB6*	NADH dehydrogenase [ubiquinone] 1 beta subunit 6	86.31	38	3
W5Q1B0	ENSOARP00000016496	*NDUFB8*	NADH dehydrogenase [ubiquinone] 1 beta subunit 8	64.26	15	2
W5PGA3	ENSOARP00000009469	*NDUFB9*	NADH dehydrogenase [ubiquinone] 1 beta subunit 9	98.66	16	3
W5Q5Y9	ENSOARP00000018129	*NDUFB10*	NADH dehydrogenase [ubiquinone] 1 beta subunit 10	109.34	28	4
W5P9Q8	ENSOARP00000007167	*NDUFC2*	NADH dehydrogenase [ubiquinone] 1 subunit C2	88.79	20	2
W5PJU2	ENSOARP00000010713		NADP oxidoreductase domain	113.31	22	5
W5PJJ7	ENSOARP00000010618		NADP oxidoreductase domain	110.5	12	5
Complex II- Succinate dehydrogenase (EC 1.3.5.1)						
W5Q216	ENSOARP00000016754	*SDHA*	Succinate dehydrogenase	203.03	31	18
W5PNR7	ENSOARP00000012093	*SDHB*	Succinate dehydrogenase	109.59	25	7
Complex III- Cytochrome c reductase (EC 1.10.2.2)						
W5Q0F9	ENSOARP00000016195	*UQCRC2*	Cytochrome b-c1 complex subunit 2	274.48	55	30
W5Q5G6	ENSOARP00000017956	*UQCRC1*	Ubiquinol-cytochrome c reductase	272.33	50	33
W5PUP9	ENSOARP00000014181	*UQCRQ*	Ubiquinol-cytochrome c reductase	123.12	30	4
W5P642	ENSOARP00000005896	*UQCRB*	Ubiquinol-cytochrome c reductase	130.16	50	5
W5PZC9	ENSOARP00000015815	*UQCRH*	Cytochrome b-c1 complex subunit 6	98.94	57	3
W5P6B2	ENSOARP00000005966	*UQCR10*	Ubiquinol-cytochrome c reductase	92.15	44	3
W5P2X9	ENSOARP00000004779	*UQCRFS1*	Cytochrome b-c1 complex subunit Rieske	156.71	41	10
C9E8M7	ENSOARP00000005022	*CYB5A*	Cytochrome b5 type A	52.55	29	2
W5P066	ENSOARP00000003814	*CYB5B*	Cytochrome b5 type B	116.91	41	6
W5QCI3	ENSOARP00000020430	*CYB5R3*	Cytochrome b5 reductase 3	134.01	26	5
W5Q0A9	ENSOARP00000016145	*CYC1*	Cytochrome c1	180.21	36	10
P62896	ENSOARP00000022795	*CYC*	Cytochrome c	115.32	27	3
Complex IV- Cytochrome c oxidase (EC 1.9.3.1)						
O78750	ENSOARP00000000004	*COX2*	Cytochrome c oxidase subunit 2	101.8	30	4
W5PPE8	ENSOARP00000012324	*COX4I1*	Cytochrome c oxidase subunit 4I1	172.49	41	11
W5NXT8	ENSOARP00000002985	*COX5A*	Cytochrome c oxidase subunit 5A	187.64	73	15
W5PW27	ENSOARP00000014660	*COX5B*	Cytochrome c oxidase subunit 5B	142.11	73	9
W5P4E7	ENSOARP00000005299	*COX6B1*	Cytochrome c oxidase subunit 6B1	169.46	72	14
W5P473	ENSOARP00000005225		Cytochrome-c oxidase activity	86.89	53	4
W5PC47	ENSOARP00000019109		Cytochrome-c oxidase activity	96.35	59	5
W5PNG6	ENSOARP00000011992		Cytochrome-c oxidase activity	82.17	28	3
W5Q6E1	ENSOARP00000018281		Cytochrome-c oxidase activity	86.89	52	4
W5PXG3	ENSOARP00000016418		Cytochrome-c oxidase activity	81.29	36	4
Complex V- ATP Synthase (EC 3.6.3.10 and EC 3.6.3.14)						
W5P471	ENSOARP00000005223	*ATP4A*	ATPase H^+^/K^+^ transporting subunit 4A	49.23	6	4
W5QEA9	ENSOARP00000021057	*ATP5F1*	Hydrogen ion transmembrane transporter activity	65.12	10	3
W5Q5U7	ENSOARP00000018087	*ATP5L*	Hydrogen ion transmembrane transporter activity	84.63	28	3
W5NY50	ENSOARP00000003098	*ATP5A1*	ATP synthase H^+^ transporting subunit 5A1	181.52	29	14
W5PEP7	ENSOARP00000008911	*ATP5B*	ATP synthase H^+^ transporting subunit 5B	236	46	25
W5PP37	ENSOARP00000012213	*ATP5H*	ATP synthase H^+^ transporting subunit 5H	153.27	38	6
W5PF18	ENSOARP00000009033	*ATP5I*	ATP synthase H^+^ transporting subunit 5I	42.72	39	2

Uniprot mammalia (Uniprot No.) and Ensembl ovine accession numbers (Ensembl No.), gene and protein name, probability score (−10log*P*), number of peptides matched (Pep.) and sequence coverage (Cov. %) are shown

### Mitochondrial associated membranes (MAM)

Several of the proteins identified play a role in interactions between organelles such as the ER and MAM proteins or chaperones [35, 36]. These include voltage-dependent anion-selective channel protein (VDAC1, VDAC2 and VDAC3; Additional file 1) and chaperones 78 kDa glucose regulated proteins (HSPA9 and HSPA5), calnexin (CANX) and ER resident protein 44 (Erp44) also known as protein disulfide-isomerase (PDIA).

### Calcium homeostasis and calcium binding proteins

Proteins identified that play a role in intracellular Ca^2+ ^regulation and balance (Additional file 1) containing Ca binding motifs called EF-hands were found. These occur in S100 Ca-binding proteins that are thought to be important in the process of terminal differentiation through transglutaminase activity that is Ca dependent. We identified 5 S100 (S100A2, S100A8, S100A9, S100A11, and S100A12) and a number of annexins (AXNA1 to 7 and AXN11) known to interact with S100 proteins. All annexins are Ca and phospholipid binding proteins. Other proteins that bind Ca in the ER were found and include calreticulin and calnexin.

### Regulators of cell growth and metabolism (cytokines)

We detected epidermal growth factor receptor (EGFR; Table 4) that binds epidermal (EGF) and transforming growth factor alpha (TGFα) or ligands that contain an EGF-like domain. Two proteins that we identified with EGF-like domains were milk fat globule-EGF factor 8 protein, also called lactadherin (MFGE8; 2 EGF domains) and transforming growth factor-beta-induced protein Ig-h3 (TGFβI; 16–18 EGF repeats).

**Table 4:** A summary of proteins identified that have a biological functions to regulate growth, differentiation and cell signalling as a cytokine or steroid

Uniprot No.	Ensembl No.	Gene name	Protein name	-10log*P*	Pep.	Cov., %
W5PWC5	ENSOARP00000014758	*EGFR*	Epidermal growth factor receptor	153.07	10	9
W5Q0F3	ENSOARP00000016189	*TGFBI*	Transforming growth factor beta induced protein	163.72	28	11
W5PNP1	ENSOARP00000012067	*MFGE8*	Milk fat globule-EGF factor 8 protein	219.51	45	21
W5NS44	ENSOARP00000000984	*HSD17B12*	Hydroxysteroid 17-beta dehydrogenase	120.1	18	4
W5P446	ENSOARP00000005198	*HSD17B13*		236.93	59	21
W5NRH5	ENSOARP00000000765	*HSD17B4*		140.67	14	7
W5PE74	ENSOARP00000008738	*NSDHL*	NAD(P) dependent steroid dehydrogenase	80.61	5	2
W5Q7P4	ENSOARP00000018736	*PIP*	Prolactin induced protein	110.58	37	5
W5Q0U6	ENSOARP00000016332	*PGRMC2*	Progesterone receptor membrane component 2	114.48	25	5

Accession number for Uniprot mammalia and Ensembl *Ovine aries* database, gene name, protein name, probability score (−10log*P*), number of peptides matched (Pep.) and sequence coverage (Cov. %) are shown. Details of the protein identifications are found in Additional file 1: Table S1

We also identified a component of the progesterone-binding protein complex, the membrane-associated progesterone receptor component 1 (PGRMC1) which can bind progesterone. Additionally, we also detected clathrin heavy chain (CLTC). Clathrin assists in the formation of coated pits and vesicles involved in intracellular trafficking of cytokine receptors as well as in endocytosis of a variety of macromolecules through remodelling in the plasma membrane.

### Proteins with other biological functions

Proteins involved in detoxification identified include thiosulfate sulfurtransferase (TST) and glutathione S-transferase (GSTP1, and MGST3). Several antioxidant proteins such as peroxiredoxins (PRDX1–3, and PRDX6) and thioredoxin (TXN) or oxidative stress glutathione reductase (GSR, mitochondrial) were also identified.

Retinoic acid can be produced by the conversion of retinol to retinoic acid. Retinoic acid is the major physiologically active form of vitamin A and is known to regulate the expression of different genes. We identified three enzymes that generate retinoic acid including aldehyde dehydrogenases (ALDH2, Aldh1a7; Additional file 1) and retinal dehydrogenase 1 (ALDH1A1). Downstream of these processes are the UDP-glucuronosyltransferase that can detoxify steroids and fatty acid derivatives such as retinoids or bile salts through the process of glucoronidation. Several UDP-glucuronosyltransferases (UGT1, UGT2B7 and UGT2B4) known to function in this way were identified.

Other proteins of note found associated with nuclear or DNA processing were histones (H2AFX, HIST1H2BD, BG, BN, BI, H3F3A, H3PF3C, HIST1H4F, 4H, 4I), 5′-nucleosidase (NT5E) and purine nucleoside phosphorylase (PNP).

## Discussion

The procedure developed has expanded our knowledge of the epithelium proteins involved key cellular processes. This new procedure provides a more specific coverage of the rumen epithelium proteome than previously reported [20–22]. In these studies, proteins were profiled while the epithelium and LP was attached. Although, [22] identified higher numbers of proteins (*n* = 813) than we did, the diet of the animals, which has a marked effect on rumen epithelium, was not defined and the representation of keratin proteins was surprisingly low (6%). Previously the separation of the epithelium from the underlying LP has only been attempted by laser capture microdissection for gene expression assays [37]. Our successful use of dispase II for enzymatic separation of tissue layers indicates the basement membrane between the *S. basale* to the LP contains fibronectin and collagen IV, not dissimilar to the skin epidermis. Here we present the highlights of our findings using the procedure to isolate the epithelial proteins.

Our results represent the epithelium proteins exposed to a fibrous diet containing 90% DM, 16.3% CP, 50.6% NDF and 9.9 MJ ME /kg DM. In general, the ratio of acetate and butyrate to propionate is greater on a roughage-based diet [38] than a concentrate diet. Unlike other reports of goats fed low, medium and high energy diets [21] or dairy sheep fed a concentrate supplemented diet [22], the fibrous diet we fed was accompanied by the presence of hard keratins in rumen epithelium. These have previously been found in hair, hoof or horn. Since intermediate filaments perform the function of responding to external forces, this is not surprising. Our result validates previous reports on gene expression in full thickness rumen wall tissue of sheep fed different quality fibrous diets [1]. They reported the gene expression of KFAP and the cornified envelope genes such as small proline-rich proteins in rumen tissue. It is possible that the extraction procedure we used did not solubilise some of these proteins as it has been found that different concentrations of urea are required to solubilise KFAP [39].

Our evidence reveals that the surface layer of the epithelium appears to contain hardened cornified epithelium that would be expected to form a barrier to nutrients and ions. The tight junctions formed between the cells that are thought to affect the paracellular permeation of small molecules to basal layers of the epithelium. Proteins contributing to cell-cell attachment such as desmosome and hemi desmosome plaques were identified.

Subunits of active transporter Na^+^/K^+^ -ATPase’s indirectly involved in nutrient transport were detected which is a membrane protein with up to 10 TMHMM. It is surprising that some of the previously reported membrane bound transporter of SCFA, lactate and pyruvate, such as SLC16 [40], were not detected using our method despite profiling a membrane fraction. Additionally, membrane bound transporters of proteins and nitrogenous compounds such as aquaporin’s [12] and peptides SLC15A1 [13], or Na^+^-glucose cotransporters (SLC2A1; SGLT-1) [41] were not identified. It is likely other proteins motifs were not detected because of their low abundance, as only 8% of the proteins identified had a subcellular assignment to the plasma membrane or were associated with organelle membranes. Presumably using more sensitive technologies with a data dependent acquisition approach and the use of ion spectral libraries such as the SWATH-MS technique will improve the coverage of these proteins.

Our procedure confirmed previous findings about the major biological functions carried out in the epithelium [1, 18]. These include the metabolism of long chain FA and SCFA, peptides and a relatively small proportion of monosaccharides through the citric acid cycle, that form acetyl CoA and ketone bodies such as acetoacetate and β-hydroxybutyrate. A proportion of ketone bodies and SCFA may be absorbed into the bloodstream of the animal or provide cellular energy in the form of ATP through oxidative phosphorylation in the mitochondria. Many of the proteins involved in the electron transport chain were also identified. Some of these are described [22] in bovine rumen tissue protein profiles. Other processes involved calcium homeostasis or binding and regulation of intracellular oxidative stress including the functions associated with MAM.

Those proteins that play a key role in epithelial biology in other tissue such as skin or the upper digestive tract were identified. EGFR is one such cytokine receptor that has been reported in transcript studies in the rumen wall in dairy cows [42]. The stimulatory effect of EGF via its receptor on cell growth has been shown in isolated rumen epithelium cell cultures [43]. Identification of EGFR and several proteins with EGF repeats indicates that these cytokines are likely involved in the regulation of growth and differentiation of the rumen epithelium, probably in the basal cells. Our identification of TGFβ induced protein (TGFβ1) is novel in rumen epithelium. This protein has been found to be secreted, induced by TGFβ and associated with normal skin and adhesion of dermal fibroblasts [44] or keratinocytes [45]. In a study of calf rumen epithelium development during weaning, TGFβ1 was found to be an important transcriptional regulator of gene expression networks on certain diets [46]. Little is known about the exact function of this cytokine in adult tissue, which presents an opportunity for a more thorough exploration of its function in the rumen epithelium. Other cell specific factors that have diverse regulatory functions in cells, were enzymes associated with prostaglandin and arachidonic acid metabolism.

Our procedure allowed the identification of proteins involved in nutrient absorption and trafficking transported through integral membrane proteins and possibly endocytosis. In particular, those that form the barrier function and formation of cell-cell junctions were identified. Many of the proteins involved with the electron transport chain apparatus were identified, as were key enzymes involved in ketone body formation. In combination with transcriptome studies, a comprehensive understanding of cellular function and gene networks operating in the rumen epithelium will emerge.

## Conclusion

Our research has defined proteins specific to the rumen epithelium of sheep. We identified intermediate filaments typical of basal and cornified epithelium. Cytosolic and membrane proteins including those of cellular organelles were identified. In addition to structural, nutrient transport and metabolism proteins, those that regulate cell growth, differentiation and signalling are reported.

## Additional file

Additional file 1: Categories and characterisation of all proteins identifiied in the rumen epithelium proteome. (XLSX 186 kb)

## Abbreviations

ALC: Average local confidence
ATP: Adenosine triphosphate
Ca^2+^: Calcium
CBB: Coomassie brilliant blue
CH_3_CN: Acetonitrile
Cl^−^: Chloride
CP: Crude protein
Da: Dalton
DM: Dry matter
DTT: Dithiothreitol
EGF: Epidermal growth factor
ER: Endoplasmic reticulum
FDR: False discovery rate
GPI: Glycosylphosphatidylinositol
H^+^: Hydrogen
HCO_3_^−^: Bicarbonate
IDA: Information dependent acquisition mode
IF: Intermediate filaments
K^+^: Potassium
KAP: Keratin associated proteins
LC MS / MS: Liquid chromatography tandem mass spectrometry
LCE: Late cornified envelope
LDS: Lithium dodecyl sulfate
LP: Lamina propria
m/z: Mass to charge ratio
MAM: Mitochondrial associated membranes
MJ ME /kg DM: Megajoules of metabolisable energy per kg of dry matter
MWCO: Molecular weight cut off
Na^+^: Sodium
NADH: Reduced nicotinamide adenine dinucleotide
NDF: Neutral detergent fibre
PBS: Phosphate buffered saline
RFI: Residual feed intake
*s*.: Stratum
SCFA: Short chain fatty acids
SDS: Sodium dodecyl sulfate
SignalP: Signal peptide
SLC family: Solute carrier protein family
TGFα: Transforming growth factor alpha
TMHMM: Transmembrane proteins helices based on hidden Markov model
Tris: Tris (hydroxymethyl) aminomethane
β: Beta
